# The Repertory

**Published:** 1889-12

**Authors:** 


### THE REPERTORY.

It is the purpose of the editor of the Repertory (two parts of which will be published in this volume of THE HOMŒOPATHIC PHYSICIAN) to continue the publication of the Repertory in parts of about seventy-five or a hundred pages each. These parts will be published separately from this journal, and will be sold to subscribers at a special price, varying with the size of the part. Due notice will be sent to each subscriber as each part is published. The only cause for delay in the appearance of this Repertory will be the editor's inability for continued work upon its revision.

